# Rapid analyses of dry matter content and carotenoids in fresh cassava roots using a portable visible and near infrared spectrometer (Vis/NIRS)

**DOI:** 10.1371/journal.pone.0188918

**Published:** 2017-12-11

**Authors:** Ugochukwu N. Ikeogu, Fabrice Davrieux, Dominique Dufour, Hernan Ceballos, Chiedozie N. Egesi, Jean-Luc Jannink

**Affiliations:** 1 Plant Breeding and Genetics Section, Cornell University, Ithaca, NY, United States of America; 2 National Root Crops Research Institute, Umudike, Nigeria; 3 Centre de Coopération Internationale en Recherche Agronomique pour le Développement (CIRAD), UMR Qualisud, St. Pierre, Reunion Island, France; 4 Centre de Coopération Internationale en Recherche Agronomique pour le Développement (CIRAD), UMR Qualisud, Montpellier, France; 5 Centre de Coopération Internationale en Recherche Agronomique pour le Développement (CIRAD), UMR Qualisud, Cali, Colombia; 6 Centro Internacional de Agricultura Tropical (CIAT), Apartado Aéreo 6713, Cali, Colombia; 7 International Institute of Tropical Agriculture (IITA), Ibadan, Nigeria; 8 United States Department of Agriculture (USDA), Ithaca, NY, United States of America; Agricultural University of Athens, GREECE

## Abstract

Portable Vis/NIRS are flexible tools for fast and unbiased analyses of constituents with minimal sample preparation. This study developed calibration models for dry matter content (DMC) and carotenoids in fresh cassava roots using a portable Vis/NIRS system. We examined the effects of eight data pre-treatment combinations on calibration models and assessed calibrations on processed and intact root samples. We compared Vis/NIRS derived-DMC to other phenotyping methods. The results of the study showed that the combination of standard normal variate and de-trend (SNVD) with first derivative calculated on two data points and no smoothing (SNVD+1111) was adequate for a robust model. Calibration performance was higher with processed than the intact root samples for all the traits although intact root models for some traits especially total carotenoid content (TCC) (R^2^_c_ = 96%, R^2^_cv_ = 90%, RPD = 3.6 and SECV = 0.63) were sufficient for screening purposes. Using three key quality traits as templates, we developed models with processed fresh root samples. Robust calibrations were established for DMC (R^2^_c_ = 99%, R^2^_cv_ = 95%, RPD = 4.5 and SECV = 0.9), TCC (R^2^_c_ = 99%, R^2^_cv_ = 91%, RPD = 3.5 and SECV = 2.1) and all Trans β-carotene (ATBC) (R^2^_c_ = 98%, R^2^_cv_ = 91%, RPD = 3.5 and SECV = 1.6). Coefficient of determination on independent validation set (R^2^_p_) for these traits were also satisfactory for ATBC (91%), TCC (88%) and DMC (80%). Compared to other methods, Vis/NIRS-derived DMC from both intact and processed roots had very high correlation (>0.95) with the ideal oven-drying than from specific gravity method (0.49). There was equally a high correlation (0.94) between the intact and processed Vis/NIRS DMC. Therefore, the portable Vis/NIRS could be employed for the rapid analyses of DMC and quantification of carotenoids in cassava for nutritional and breeding purposes.

## Introduction

Near infra-red spectroscopy (NIRS) is one of the most important analytical techniques based on the vibrational properties of atoms in molecules [1,2]. NIRS has gained wide application over years in the analyses of many materials including agricultural and food products [3,4]. When compared to other analytical and chemical methods, NIRS offers a fast, non-destructive alternative for the simultaneous analyses of many constituents [5]. It requires minimal to no sample preparation, and it is economically efficient and non-hazardous to the environment [6].

NIRS is an ideal phenotyping tool in plant breeding, particularly in this era when new breeding techniques are being adopted [7,8], requiring the phenotyping of thousands of individuals at low cost and with high precision and speed. NIRS permits the timely screening of many samples and variables that would have been too expensive to assay by other analytical methods [8,9]. One of its notable advantages, is its ability to measure samples in different states–in solid and liquid forms [10].

Breakthroughs in technology have led to the increasing availability of spectrophotometers of different ranges in a portable format and this provides greater flexibility for field-based analyses of constituents. The portable NIRS and in some cases covering both the visible and near infrared regions (Vis/NIRS) has the advantage of further reducing the need for sample transportation to a laboratory and processing. It provides a quality phenotyping method for breeding programs especially where standard laboratories are not available or their operation is hampered by factors such as poor infrastructure and lack of highly skilled experts. It is believed [6] that over the long-term developing NIRS is cheaper than the establishment of many protocols for laboratory analyses of different traits, which in most cases are slow, costly and impractical for large-scale screening in plant breeding and nutritional quality analyses [7,11].

In cassava breeding, the adoption of new methods has necessitated standardized and accurate phenotyping tools for efficient improvement, especially for complex traits [12]. Availability of phenotyping tools for accurate and large scale screening of materials, particularly at early stages of cassava breeding will reduce the loss of important genetic information and facilitate the breeding of end-user and farmer-preferred varieties [13]. The current phenotyping techniques for some key traits are laborious and time-consuming for large-scale screenings. Estimates could be influenced by sampling and sample preparation including weight and number of roots used in the prevalent specific gravity method [14–16] and inconsistency of power supply in the oven-drying method. Similarly, carotenoid quantification using color intensity [17] could be subjective and inefficient in advanced population of yellow genetic materials. Conversely, laboratory processes using high-performance liquid chromatography (HPLC) or UV-Visible spectrophotometer are low-throughput (less than 10 or 40 samples per day, respectively) [11].

The use of NIRS for the analyses of traits on fresh cassava roots have been previously reported [11] and has led to significant changes in a breeding system [18]. However, these studies used a stationary tabletop NIRS device with processed root samples–peeled and mashed, aimed at overcoming the reported uneven concentration of traits in cassava roots [19]. Nevertheless, the possibility of reduced sample preparations using intact samples have been reported in other scenarios [20–22]. Preparation of cassava root samples before NIRS analysis adds to the harvesting time and the overall cost of phenotyping. The use of a full-range portable Vis/NIRS device has not been reported in cassava breeding and the possibility of reduced root processing has not equally been explored. Obtaining a good relationship between calibrations from processed and intact samples could enable simultaneous field-based screening of materials on different important traits and the overall reduction of phenotyping cost.

Generally, when working with NIRS, the spectral variation of interest can be masked by additive and/or multiplicative light scattering, background noise and baseline drifts arising from differences in particle sizes and effective path-length [23,24]. It is therefore important to adopt suitable data pre-processing methods to minimize the influence of these physical effects on the NIRS calibration [24,25].

In this study, we assess the use of a portable Vis/NIRS device for the analysis of important fresh cassava quality traits on both processed (mashed) and non-mashed (intact) root samples. We assess the impact of data pre-processing for possible increase in the predictive ability of the calibration models. The ultimate goal of this study was to develop calibration models using the portable Vis/NIRS for the analyses of DMC and carotenoids in fresh cassava roots which could accelerate accurate phenotyping and general improvement of cassava. To examine the usefulness of this tool on dry matter quantification, we compared dry matter values derived from the conventional specific gravity method and predicted values from the portable Vis/NIRS (intact and mashed) to the ideal oven-drying method.

## Materials and methods

### Calibration samples

In 2015, first calibration set (Table 1) was developed using clones (U15I, N = 113) from the germplasm collection of the National Root Crops Research Institute (NRCRI), Umudike, Nigeria. Single root samples were randomly selected from harvested clones of a training population (TP) established for the implementation of genomic selection. The selected roots were peeled and chopped into pieces (about 3x10 mm) using kitchen knives.

**Table 1:** Description of calibration sets developed at NRCRI Umudike, Nigeria and CIAT, Cali Colombia in 2015 and 2016 on intact and mashed root samples. Carotenoids (TCC and ATBC) data are on a fresh weight basis.

Statistics	U15I	C16I	U16I/M	C16M	U15I	C16I	C16M	C16M
	DMC (%)				TCC (μg g^-1^)			ATBC (μg g^-1^)
No.	113	66	194	173	113	65	173	173
Mean	35.75	20.14	38.52	36.16	2.61	17.95	14.91	10.07
SD	7.95	4.27	5.76	4.16	2.14	3.84	7.73	5.86
Min.	16.34	16.54	16.47	20.14	0.10	10.09	0.70	0.03
Max.	50.98	41.98	50.00	44.13	8.82	26.15	30.84	21.02

U15I = Calibration set on intact root samples at Umudike in 2015; U16I/M = Calibration set on intact and mashed roots at Umudike in 2016; C16I = Calibration set on intact roots from CIAT in 2016; C16M = Calibration set on mashed roots from CIAT in 2016.

A second calibration set (Table 1) was developed in 2016 at the International Center for Tropical Agriculture (CIAT), Cali-Palmira, Colombia. Between two to three root samples were collected from F1 seedling plants of different half- and full-sib families of varying sizes [18,26]. Additional clones with white parenchyma from the germplasm collection at CIAT were added in order to balance the calibration set. All the field sampling and selections were carried out early in the morning and the selection of individuals for carotenoid was based on yellow/orange color intensity of roots which is closely associated with high carotenoids especially TCC and total beta carotene (TBC) in cassava [11,17]. The selected roots were peeled and mashed into a homogenous sample in the laboratory using an Essen Skymsen food processor (Model: PA-7SE, Brusque, Brazil).

A third calibration set (Table 1) was developed in 2016 at NRCRI for DMC using intact and mashed root samples. Between two to three roots were randomly selected from one or two plants in a plot of five plants per clone from the NRCRI TP. The selected roots were evaluated for DMC by specific gravity before peeling and mashing using a portable power-operated grater.

The 2016 set from NRCRI and a subset of the calibration set from CIAT were used for the comparison of calibrations from intact and mashed root samples.

### Spectral data collection

A portable Vis/NIRS device (QualitySpec Trek: S-10016) was used to collect spectral data on both intact and mashed root samples. Spectral data on intact roots were obtained by placing roots in contact with the window of the portable Vis/NIRS device. Each spectrum collected is in fact the average of 50 spectra collected over a period of five seconds. Three spectra per root were taken respectively on the proximal, middle and distal regions of roots at NRCRI in 2016 and CIAT. The selected root samples were first peeled, rinsed with water and dried with a paper towel before spectra collection. However, depending on the size of the roots, spectral data were only collected from the transverse section of the proximal end of the root and few samples from proximal and distal ends in 2015 at NRCRI. The mean spectrum for each sample was used for calibration.

For mashed samples, spectral data were collected from about 8g of homogenized mashed roots in quartz sampling cups placed against the window of the portable Vis/NIRS device in two replications per sample and spectrum averages were used for analyses.

### Wet chemistry

#### Dry matter content (DMC)

At both locations (CIAT and NRCRI), dry matter was measured as the percentage of dry weight relative to a given fresh weight of samples after oven-drying. Between 80 and 110 g (measured to 0.1 mg precision) of the mashed and homogenized roots were oven-dried at a constant temperature of 105°C for 24 hours at CIAT. At NRCRI in 2015, 10 g of the chopped samples were weighed before and after oven-drying while in 2016, 20 g of the mashed samples were dried in two replications. The oven temperature at NRCRI was targeted for TTT°C. Depending on the duration and source of power, samples were weighed after drying. The average DMC of the two replications was used for analyses. Specific gravity method as described in [14] was carried out before the selected two to three roots were processed–peeled, washed and dried with a paper towel in 2016 at NRCRI.

#### Carotenoids

The reference samples at CIAT were measured for carotenoids using a HPLC system (Agilent Technologies 1200 series, Waldbronn, Germany). To avoid quality degradation of samples, an average of six (6) samples per day were analyzed with the HPLC. Similar to [11] and complying with the HarvestPlus standards for optimum carotenoids retention [27], all the extractions were performed on fresh roots with minimal exposure to light, high temperatures and reduction of time between mashing and extraction. The HPLC reference traits included–TCC, all-trans β-carotene (ATBC), violaxanthin (VIO), Lutein (LUT), 15-Cis beta-carotene (15CBC), 13-Cis beta-carotene (13CBC), Alpha carotene (AC), 9-Cis beta-carotene (9CBC) and phytoene (PHY).

Measurement of TCC at NRCRI in 2015 was carried out at the NRCRI Carotene laboratory in Umudike following the standard laboratory extraction method using acetone with mortar and pestle and spectrophotometric quantification as described in the Harvest-Plus handbook [27]. Homogenized samples of 10g were ground in a mortar with 3g of Hyflosuperce (Celite) and 50mL of cold acetone. The mixture was filtered with a Buchner funnel with filter paper while the mortar, pestle, funnel and residue were washed into a suction flask and observed to be sure that the washings or residue were devoid of color. Otherwise, the residue was returned to the mortar for further maceration, filtering and washing. The next step involved the petroleum ether partitioning where about 20mL of petroleum ether and acetone were added into a 500mL separator funnel with Teflon stop-cock. Distilled water (~300mL) was slowly added into the mixture. The two phases were allowed to separate and the lower, aqueous phase was discarded while the remaining phase was washed 3–4 times with distilled water (~200mL) to remove residual acetone. The petroleum ether phase was transferred into a 25mL volumetric flask through a funnel containing glass wool and anhydrous sodium sulphate (about 15 g) to remove the residual water. The absorbance of the extract was measured at 450 nm using a spectrophotometer (Electron Corporation Ltd–GENESYS 10 Series) and TCC was derived using:

$$\begin{array}{l} TCC\;(\mu /g)=\frac{A\times Volume\;(mL)\;\times 10^{4}}{A_{1cm}^{1\%}\times sample\;weight\;(g)},\\ \;\;where,\;A=\mathrm{absorbance};\;Volume=\mathrm{total}\;\mathrm{volume}\;\mathrm{of}\;\mathrm{extract};\;A_{1cm}^{1\%}\\ \;=\mathrm{absorption}\;\mathrm{coefficient}\;\mathrm{of}\;\beta -\mathrm{carotene}\;\mathrm{in}\;\mathrm{Petroleum}\;\mathrm{ether}\;(\mathrm{equals}\;2592). \end{array}$$

### Data pre-processing and model development

Prior to model development, spectral data were first transformed to log (1/R) using ViewSpec Pro software [28] and the full Vis/NIRS wavelength range (350 – 2500nm) was subjected to pre-treatments for the correction of interferences on three segments of the wavelengths (350nm -1000nm, 1001nm– 1800nm and 1801nm– 2500nm). The effect of two light-scatter correction methods—Standard Normal Variate and De-trending (SNVD) [29] and Multiplicative Scatter Correction (MSC) [30] were tested on four derivative and smoothing options. The options are given by four digits (D, G, S1, S2): where D indicates the derivative order number (0 indicates no derivation, 1 means the first derivative, and so on), G indicates the gap (the number of data points over which derivation is computed), S1 indicates the number of data points in the first smoothing (1 means no smoothing) and S2 indicates the number of data points in the second smoothing, where 1 means no smoothing. The eight pre-treatment methods (SNVD+1111, SNVD+2111, SNVD+1551, SNVD+2551, MSC+1111, MSC+2111, MSC+1551 and MSC+2551) were compared to no treatment in each calibration set for DMC, TCC and ATBC.

#### SNVD

The SNVD correction requires two algorithms that are usually applied together. The first algorithm is the Standard Normal Variate (SNV) and is used for correcting scattering when the effective path length and baseline varies among samples of a data set [23] and for granular or powdery samples or when the particle sizes vary among samples [29]. SNV is usually applied first to correct the effects of the multiplicative interferences of scatter and particle size differences by removing the mean and scaling to unit variance. SNV correction is given by:

Si=(S0−Sv)Sd,whereSi=correctedspectrum,So=originalindividualspectrummeasuredbytheNIRdevice,Sv=averagevalueofthesamplespectrumtobecorrected,Sd=standarddeviationofthesamplespectrum.

De-trending attempts to remove the additional variation in baseline shift and curvelinearity by fitting the spectral values of a given *i* spectrum at *k* wavelength (*S*_*i*,*k*_) to a polynomial function–for example, a quadratic function *(`S*_*i*,*k*_*)* (Di) and subtracts the function (quadratic baseline) from the spectral values (Dii) [25]:

$$`S_{i,}k=a+b.k+c.\;k^{2}\;Di$$

$$S_{i,\;k}{}_{\left(De-trend\right)}=Si,\;k-`Si,\;k\;\mathrm{Dii}$$

SNVD does not require external references and each spectrum is treated independently of others in the training set [24].

#### MSC

This method attempts to correct for particle size dependence by linearizing each spectrum to an ideal or reference sample spectrum which in most cases is the average spectrum obtained from all the data in the training set. The slope and offset of the sample spectra are adjusted to the ideal average spectra to give the MSC corrected spectrum [24,30]. The process of MSC correction, assuming the reference is the mean, includes:

1. Reference spectrum calculation: ${\bar{S}}_{j}=\sum_{i=1}^{n}(S_{i,j})/n$
2. Using spectral responses in each spectrum to calculate a linear regression against the corresponding points in the reference spectrum: $S_{i}=a_{i}\bar{S}+b_{i}$
3. Subtracting the slope from the regression on the original spectrum and dividing with the offset values to obtain MSC corrected spectrum:
  $$\begin{array}{l} S_{i\left(MSC\right)}=(S_{j}-\;b_{i})/\;a_{i},\;where\;S=spectral\;responses\;for\;all\;the\;wavelengths;\;\bar{S}\\ =average\;responses\;of\;all\;the\;training\;set\;spectra\;at\;each\;wavelength;\;S_{i}\\ =responses\;for\;a\;\mathrm{singl}e\;spectrum\;in\;the\;training\;set;\;n\\ =number\;of\;training\;spectra;\;a_{i}\;and\;b_{i}\\ =slope\;and\;offset\;coefficients\;of\;the\;linear\;regression\;of\;the\;mean\;spectrum\;vector\;\bar{S}\;versus\;S_{j}\;spectrum. \end{array}$$

#### Derivatives and smoothing

The basic method of derivation is finite difference where: the first-order derivation, takes the difference between two values with a given gap size while second order derivative is then estimated by calculating the difference between two successive points of the first-order derivative spectra [24,31]. The basic derivative is usually not feasible for most real measurements due to noise inflation and the modified smoothing and derivative of Norris-Williams approach [24] is used:

1. Smooth the spectra. Average over a given number of points.
  $$x_{smooth,i}=\frac{\sum_{j=-m}^{m}x_{org,i+j}}{2m+1},\;\mathrm{where}\;m\;\mathrm{is}\;\mathrm{the}\;\mathrm{radius}\;\mathrm{of}\;\mathrm{the}\;\mathrm{smoothing}\;\mathrm{window}\;\mathrm{centered}\;\mathrm{on}\;\mathrm{the}\;\mathrm{current}\;\mathrm{measurement}\;\mathrm{point}\;i.$$
2. Derive at each wavelength. For the first derivative take the difference between two smoothed values at a given gap distance and for the second-order derivative, take twice the smoothed value at point i and the smoothed value at a gap distance on either side:
  $$x_{i}^{\prime }=x_{smooth,\;i+gap}-x_{smooth,\;i-gap}$$
  $$x_{i}^{\prime \prime }=x_{smooth,i-gap}-2.x_{smooth,i}+x_{smooth,i+gap}$$

Spectra pre-treatments as well as model development were implemented in Win-ISI 4.0 software (Infrasoft International and FOSS, Hillerod, Denmark). The modified Partial Least Squares (MPLS) algorithm was used to set up a multivariate model based on the reference chemical values and the pre-treated spectra. The MPLS is a PLSR modified to scale the reference data and reflectance data at each wavelength to have a standard deviation of 1.0 [32,33]. It reduces the spectral data to a few orthogonal combinations (or factors) of absorbance that account jointly for the most spectral and reference value information [34].

### Validation of models

Models were developed using individual calibration sets across locations and years and each model was used to predict the values of other sets on either the mashed or intact root sample categories. However, because of the differences in references value standards, the major calibration set from mashed samples developed at CIAT was divided into two—calibration and validation sets (Table 2) using the *naes* calibration sampling algorithm [35]—*prospectr* package [36] in R for model development and validation. The *naes* sampling procedure usually uses cluster analysis to select calibration samples from large multivariate datasets. By retaining principal components explaining at least 99 percent of the total variance following a PCA on the spectral variables, k-means clustering (1000 iterations) was carried out on the principal component scores, with a number of clusters equal to the number of desired calibration samples (Table 2). The calibration set was constituted by drawing samples from the center of each cluster, leaving the remaining samples as validation set. This systematic sampling approach was used to ensure that the calibration set was representative of the dataset than a random sampling. The calibration set from intact roots in CIAT had small sample size and was only used to evaluate the possibility of direct unprocessed root assay. In order to perform cross-predictions in the WinISI software, the ASD spectra (350nm– 2500nm in 1nm gap) were trimmed to a range (400nm– 2500nm in 2nm gaps) compatible with the Win-ISI software.

**Table 2:** Descriptive statistics for model calibrations and independent set validations for DMC, TCC and ATBC using mashed root samples from CIAT, 2016.

Traits	Calibration set					Validation set				
	No.	Mean	SD	Min.	Max.	No.	Mean	SD	Min.	Max.
DMC	120	36.06	4.31	20.14	43.30	53	36.40	3.84	27.35	44.13
TCC	119	14.94	7.87	1.00	30.84	54	14.85	7.49	0.70	26.15
ATBC	119	9.97	5.89	0.029	21.02	54	10.29	5.85	0.31	20.33

Reported calibration statistics included the standard deviation (SD), coefficient of determination (R^2^), standard error of calibration (SEC) and standard error of cross-validation (SECV). In each model, leave-one-out cross-validation (iteratively removing one sample and predicting it using the remaining samples) was used for internal model assessment. The optimum number of PLS latent variables, which maximizes the covariance between the response and predictor variables was selected based on the minimum value of SECV. In addition, the ratio of performance to deviation (RPD = SD/SECV) as well as standard error of prediction (SEP) and standard error of prediction corrected for bias [SEP (C)] were used to evaluate the quality of the prediction models [11,37]. Unlike SEC and SECV, RPD is independent of parameter units and can therefore be compared between parameters [38].

Samples whose spectra had high Mahalanobis distance (H-outliers) with reference to the average spectrum or for which the difference between the reference and the predicted value was much higher than the standard error of cross-validation (SECV) (t-Outliers) were defined as outliers and removed in the calibration model. As suggested by [39,40], the outlier limits were set to 10 (H-outliers) and 2.5 (t-outliers). Up to three iterations of outlier identification and re-calibration [41] were allowed [11,33,38]. Some of the models were stable (no outliers detected) after one or two iterations.

All the datasets used for calibration and validation across the two locations–NRCRI and CIAT, in 2015 and 2016 and from intact or mashed samples can be assessed in the supplementary file–S1 Datasets.

### Correlation of DMC from alternative methods

To assess the relevance of the Vis/NIRS-derived DMC relative to the standard oven-drying and the conventional gravitational methods, we compared the Vis/NIRS-derived values from mashed and intact sampling with DMC from oven drying and specific gravity methods from 173 samples at NRCRI in 2016. The oven drying DMC has been described above. Specific gravity DMC is derived from the linear relationship between DMC and specific gravity (SG):

DMC = 158.3SG– 142, where SG is the ratio of weight of the sample in air to the difference between weights of the sample in air versus water.

The Pearson correlation was used to assess the relationships among the four various DMC sets–oven drying, SG-derived, mashed NIRS-derived and intact root NIRS-derived DMC. The regression between specific gravity and DMC for the selected samples was also estimated [14,15].

## Results and discussion

### Statistics of reference data

It is important to ensure adequate range and precision of traits in developing NIRS calibrations [42]. The range of the reference values for DMC on both sampling methods—intact and mashed roots was between 16% and 51% which seems applicable to many breeding programs for immediate evaluations and feasible DMC improvement (Table 1). The mean DMC at Umudike in 2015 on intact root samples (U15I) was higher than the mean of the reference data for the same trait generated at CIAT in 2016 on intact root samples (C16I) but lower than what was obtained at Umudike in 2016 on both intact and mashed (U16I/M) root samples (Table 1). The DMC of the intact/mashed (U16I/M) set from NRCRI in 2016 however, was higher than mashed samples from CIAT (C16M). The quantification approaches for TCC were different at NRCRI and CIAT but the mean TCC at CIAT was higher (17.95μg g^-1^and 14.91μg g^-1^on intact and mashed root samples, respectively) than NRCRI (2.14μg g^-1^) from only intact root samples. Varying ranges of carotenoids were obtained from the HPLC analyses for the carotenoids, although TCC and ATBC were used for most of the carotenoid analyses.

The use of the *naes* sampling algorithm enabled an even distribution of the calibration and validation sets of the mashed samples developed at CIAT in 2016 as seen in their descriptive statistics–mean, standard deviation and range (Table 2).

### Effect of pre-processing methods on calibration statistics for different calibration sets on intact and mashed root samples

Much emphasis has been laid on the need for optimum mathematical pre-treatment of spectra prior to model generation in order to minimize the impact of interferences arising from variation in particle sizes, optical path-length and crystalline forms on spectra [43]. Given that the portable Vis/NIRS has not been used in trait analyses in cassava, several pre-treatment combinations were tested in order to identify the best combination that would minimize the effect of interferences on prediction. A total of eight pre-processing combinations were assessed on the different calibration sets for different traits and from the two sampling methods–intact and mashed samples. The reported performances of the eight pre-treatment methods are based on R^2^ values for calibration (R^2^_c_) and cross-validation (R^2^_cv_) (Table 3). Usually, R^2^ of 0.50 has been classified as useful in the discrimination of concentrations, between 0.60–0.82 for screening and quantification, 0.83–0.90 is important in most applications, 0.92–0.96 is useful in most applications especially in quality assurance and above 0.98 is important for all applications [42]. Also, RPD has been used in evaluating the robustness of a model. RPD values greater than three (>3.0) has been considered sufficient; 2.0–3.0 (good); 1.5–2.0 (medium) and less than 1.5 (poor) for analytical quality in various applications [37,41,44].

**Table 3:** The effect of mathematical pre-treatments on models from different calibration sets.

Pre-trmt.	Der.& Sm.	R^2^	U15I	U16I	C16I	U16M	C16M		U15I	C16I	C16M		C16M
			DMC (%)					AV. DMC (%)	TCC (μg)			Av. TCC (μg)	ATBC (μg)
NONE	0,0,1,1	R^2^_c_	0.66	0.70	0.54	0.83	0.96	0.74	0.94	0.52	0.97	0.81	0.970
		R^2^_cv_	0.55	0.64	0.44	0.79	0.96	0.68	0.91	0.40	0.96	0.76	**0.970**
SNVD	1,1,1,1	R^2^_c_	0.91	0.90	0.96	0.96	0.99	**0.94**	0.96	0.90	0.99	**0.95**	0.987
		R^2^_cv_	0.64	0.65	0.55	0.84	0.95	**0.73**	0.90	0.67	0.93	0.83	0.945
	1,5,5,1	R^2^_c_	0.80	0.92	0.60	0.84	0.97	0.83	0.94	0.95	0.96	**0.95**	0.952
		R^2^_cv_	0.64	0.73	0.50	0.80	0.95	0.72	0.90	0.76	0.93	**0.86**	0.928
	2,1,1,1	R^2^_c_	0.81	0.85	0.60	0.93	0.97	0.83	0.91	0.89	0.98	0.93	0.982
		R^2^_cv_	0.41	0.37	0.22	0.46	0.55	0.40	0.57	0.62	0.86	0.68	0.847
	2,5,5,1	R^2^_c_	0.79	0.86	0.64	0.87	0.97	0.83	0.95	0.84	0.96	0.92	0.994
		R^2^_cv_	0.55	0.64	0.48	0.80	0.95	0.68	0.84	0.61	0.92	0.79	0.947
MSC`	1,1,1,1	R^2^_c_	0.77	0.91	0.97	0.96	0.99	0.92	0.95	0.89	0.99	0.94	**0.995**
		R^2^_cv_	0.59	0.68	0.57	0.83	0.95	0.72	0.89	0.64	0.94	0.82	0.944
	1,5,5,1	R^2^_c_	0.78	0.91	0.75	0.87	0.97	0.86	0.94	0.93	0.95	0.94	0.947
		R^2^_cv_	0.60	0.74	0.53	0.80	0.95	0.72	0.89	0.68	0.92	0.83	0.924
	2,1,1,1	R^2^_c_	0.79	0.85	0.60	0.93	0.97	0.83	0.90	0.89	0.99	0.93	0.984
		R^2^_cv_	0.41	0.41	0.22	0.46	0.56	0.41	0.57	0.62	0.86	0.68	0.852
	2,5,5,1	R^2^_c_	0.79	0.86	0.64	0.87	0.97	0.83	0.95	0.84	0.96	0.92	**0.995**
		R^2^_cv_	0.58	0.64	0.48	0.80	0.95	0.69	0.85	0.61	0.92	0.79	0.951

U15I = Calibration set on intact root samples at Umudike in 2015; U16I = Calibration set on intact root samples at Umudike in 2016; U16M = Calibration set on mashed root at Umudike in 2016; C16I = Calibration set on intact roots from CIAT in 2016; C16M = Calibration set on mashed roots from CIAT in 2016.

The average R^2^_c_ and R^2^_cv_ for DMC across the different calibration sets showed that SNVD+1111 had the highest average R^2^_c_ (94%) and R^2^_cv_ (73%), slightly higher than MSC+1111 with average R^2^_c_ of 92% and R^2^_cv_ of 72% (Table 3). The average R^2^_c_ from SNVD+1111 was also higher (95%) than MSC+1111 (94%) although the R^2^_cv_ using MSC+1111 (86%) was higher than that of SNVD+1111 (83%) for TCC calibrations. The highest average R^2^_c_ (~100%) for ATBC was obtained from MSC+1111 and MSC+2551 whereas the highest R^2^_cv_ (~95%) was obtained from SNVD (1111 and 2551) and MSC+2551. Across the three traits, overall average performance from SNVD+1111 (R^2^_c_ = 95% and R^2^_cv_ = 79%) and MSC+1111 (R^2^_c_ = 94% and R^2^_cv_ = 78%) were higher than other pre-treatments. It was observed that R^2^_c_ and R^2^_cv_ from other pre-treatment methods on individual sets were in some cases similar or even greater than values from SNVD+1111 or MSC+1111 but in all cases, performance from SNVD+1111 was still relatively high.

Compared to the no pre-treatment, the number of independent variables (spectra) used in pre-treatment evaluations often varied with the treatment methods. The average R^2^_c_ and R^2^_cv_ values from no pre-treatment for DMC and TCC calibrations were lower than the best pre-treatments from SNVD+1111 and MSC+1111. However, the R^2^_cv_ on individual calibration sets from no pre-treatment especially with the calibration set from CIAT in 2016 (C16M) was in some cases, higher than the R^2^_cv_ from any of the pre-treatment methods. For example, the highest average R^2^_cv_ (97%) for ATBC was obtained from no pre-treatment.

Percentage improvement of models arising from pre-treatments was higher using intact than mashed root samples. This could be attributed to higher levels of interference when using intact root than mashed samples.

Therefore, based on the R^2^_c_ and R^2^_cv_ performances, it seemed that the most promising pre-treatment using the Vis/NIRS device was SNVD+1111. The high performance of SNVD has been previously reported [29] for the same traits in cassava although using a different instrument and on different derivative and smoothing gaps (2,5,5,1) [11,38]. It is therefore necessary to adopt the most promising pre-treatment when working with NIRS devices.

### Calibration models on intact and mashed root samples

Given the higher measurement speed and minimum processing of root samples using intact roots, this method would be highly desirable with acceptable model performance. Higher accuracies with ground/processed samples have been obtained in similar settings [22,37] and the correlation between predictions from intact and ground samples could be high enough for routine screening purposes [20,22].

Using RPD as a calibration statistics to assess models developed from mashed and intact roots, the result showed that the RPD values for DMC from mashed samples were 2.50 and 4.32 from U16M and C16M calibrations, respectively (Table 4A). The RPD from intact root samples on both years– 2015 and 2016 at Umudike was 1.68 (Table 4B). For better comparison using the same number of clones from CIAT in 2016 from the mashed samples (C16M66) and intact samples (C16I66), the calibration from mashed samples was evidently higher than that of intact root samples (Table 4A and 4B). Similar results were obtained when using the same number of samples from NRCRI in 2016 (Result not presented). However, the R^2^_c_ of models from intact roots were still high (>86%) with R^2^_cv_ ranging from 55% to 65% (Table 4B).

**Table 4:** Calibration assessments of DMC from different calibration sets on mashed (a.) and intact (b.) root samples for DMC.

Calibration set	SEC	R^2^_c_	SECV	R^2^_cv_	SD	RPD
a. Calibrations of DMC on mashed root samples						
U16M	0.91	0.96	1.87	0.84	4.67	2.50
C16M	0.41	0.99	0.95	0.95	4.10	4.32
C16M66	0.52	0.99	1.04	0.94	4.24	4.08
b. Calibrations of DMC on intact root samples						
U15I	2.16	0.91	4.37	0.64	7.34	1.68
U16I	1.78	0.86	2.80	0.64	4.71	1.68
C16I66	0.77	0.96	2.59	0.55	3.86	1.49

**Fig 1:**
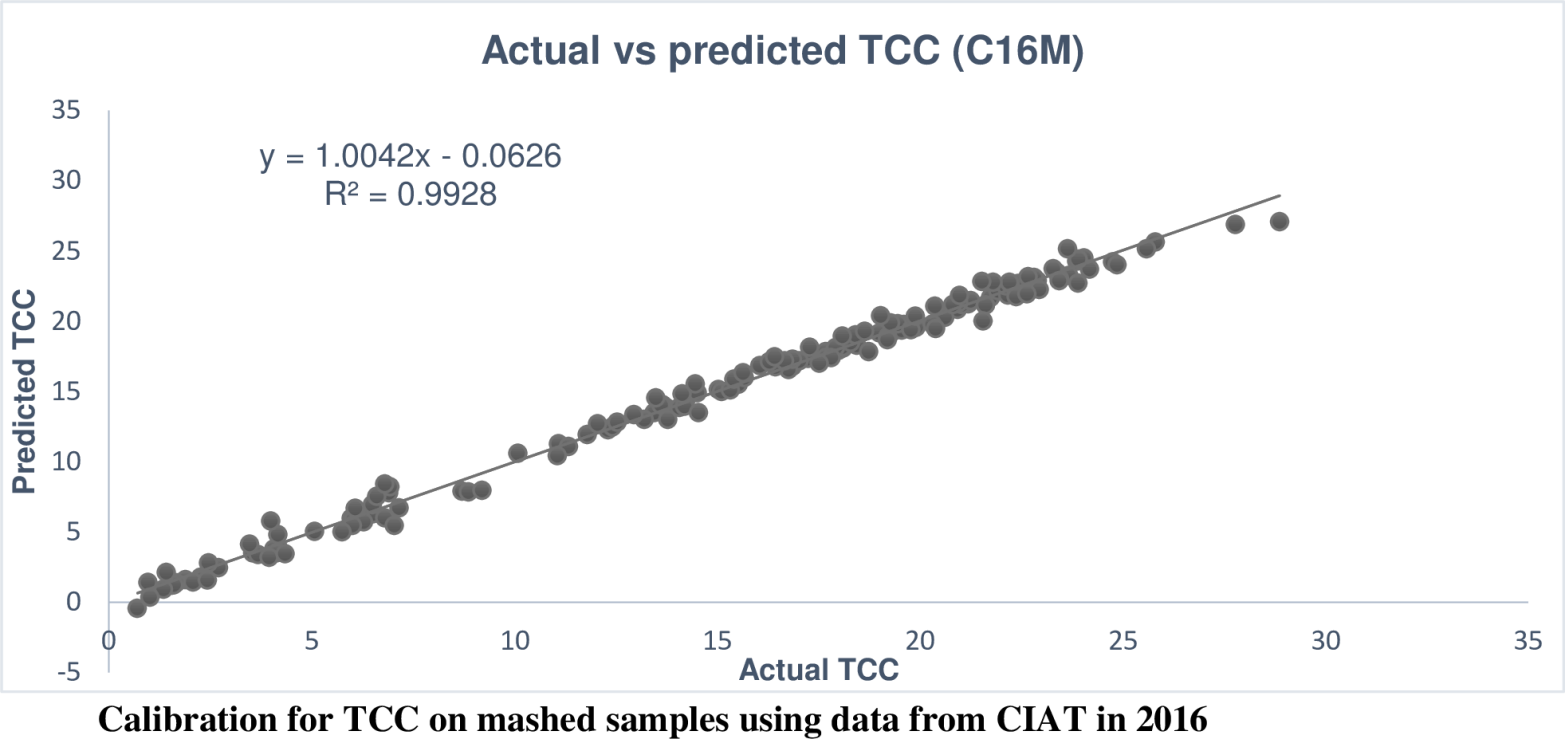
Predicted plotted against observed TCC in the calibration set, mashed samples, CIAT 2016.

**Fig 2:**
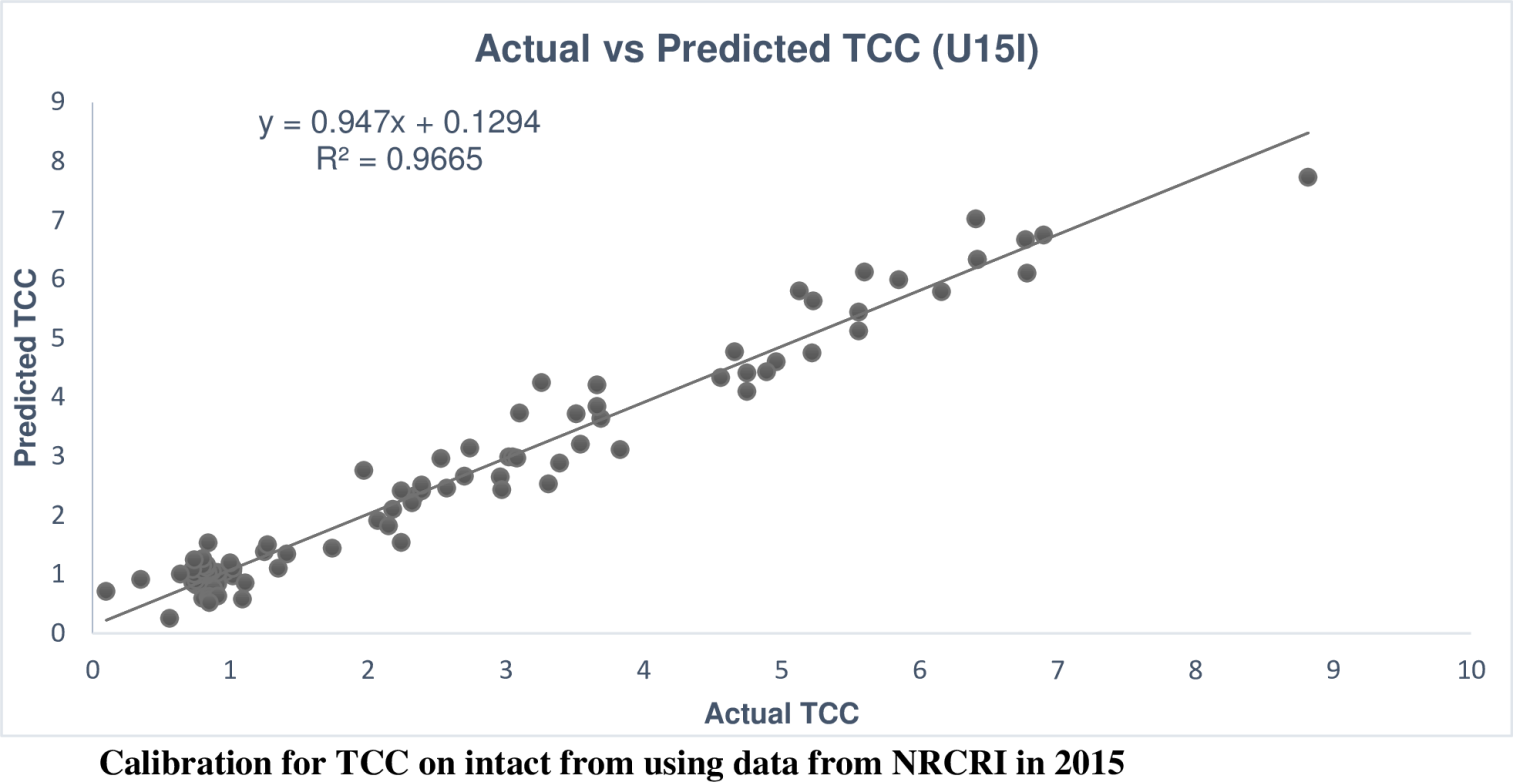
Predicted plotted against observed TCC in the calibration set, intact root samples, NRCRI 2015.

The calibration performance for carotenoids showed that the R^2^_c_ for most of the carotenoids was 99% except in alpha-carotene (80%), lutein (88%), phytoene (91%) and violin (94%), which are found at low concentration in cassava roots (S1 Table). However, the R^2^_cv_ for these traits varied from 41% in phytoene to 95% in ATBC (S1 Table and Table 5 respectively). Similar to the R^2^_cv_, the RPD was lowest in phytoene (1.31) and highest in ATBC (4.29). Comparing TCC calibration from mashed root at CIAT to TCC from intact root at NRCRI in 2015, both calibrations had very good calibration performances (Table 5A and 5B) (Figs 1 and 2). However, the calibration performance from C16M (R^2^_c_ = 99% and R^2^_cv_ = 93%; RPD = 3.79) was higher than U15I (R^2^_c_ = 96% and R^2^_cv_ = 90%; RPD = 3.16).

**Table 5:** Calibration assessments of carotenoids from mashed (a.) and intact (b.) root samples.

Cal. set	Traits (μg)	No.	Range	Mean	SD	SEC	R^2^_c_	SECV	R^2^_cv_	RPD
a. Calibration for carotenoids from mashed samples using the entire calibration set from CIAT										
C16M	TCC	164	0.70–28.87	14.84	7.32	0.64	0.99	1.93	0.93	3.79
	ATBC	161	0.03–20.33	10.05	5.53	0.64	0.99	1.29	0.95	4.29
b. Calibration for TCC using intact root samples from Umudike in 2015										
U15I	TCC	102	0.10–8.82	2.45	1.99	0.38	0.96	0.63	0.9	3.16

Similar to results obtained for DMC calibration using the same number of individuals for comparison between calibrations from mashed and intact root samples, the calibration statistics for carotenoids from mashed calibrations were still better than the calibrations from intact root (Table 6A and 6B and S2 Table). The R^2^_c_ from mashed samples varied between 73% and 99% while intact root calibrations were greater than 67% except in an extreme case where lutein was less than 50%. The R^2^_cv_ varied from 33% to 93% in mashed calibrations and 10% to 81% in intact root calibrations. Various RPD values were obtained from the two sampling methods with values from mashed roots still higher than intact root calibrations.

**Table 6:** Carotenoids calibrations from mashed (a) and intact (b) root samples from CIAT using the same sample size (n = 66).

Cal. set	Traits	No.	Range	Mean	SD	SEC	R^2^_c_	SECV	R^2^_cv_	RPD
a. Calibration of carotenoids on mashed samples										
C16M66	TCC	63	10.09–25.81	17.72	3.67	0.42	0.99	1.23	0.89	2.98
	ATBC	59	4.91–16.42	11.40	3.17	0.25	0.99	0.82	0.93	3.87
b. Calibration of carotenoids on intact root samples										
C16I66	TCC	64	10.09–26.15	17.83	3.74	1.16	0.90	2.13	0.67	1.76
	ATBC	63	4.91–19.97	11.94	3.53	0.89	0.94	1.53	0.81	2.31

Higher prediction models from ground against whole or intact samples have been reported [21,22,37] and could be attributed to higher scattering noise for spectra obtained from intact samples [21] even though the correlations between derived values from ground and intact samples are usually high [21,22]. Also, the discrepancy between models from the two sampling methods are minimal with small and less heterogeneous grains [21]. This means that reducing interferences among other things such as heterogeneity in the case of cassava [19] could improve accuracy from intact samples.

### Validation of calibration models

Validation is very important in the development of a quantitative model using independent sets of samples different from the data employed in model construction [45]. Individual models developed from different calibration sets from mashed or intact root samples were used to predict the values of other sets in the same intact or mashed sample categories. As would be expected, especially where there were obvious differences in reference value protocols, the cross-prediction statistics based on coefficient of determination (R^2^_p_) were less than 50% except in the case of using U16M for calibration and C16M for validation on DMC calibration (Table 7).

**Table 7:** Validation using different calibration sets on intact and mashed root samples for DMC.

Calibration set	Validation set	SEP	SEP(C)	R^2^
a. Cross-calibration set validations on intact root samples				
U15I	U16I	133.37	7.45	0.03
U15I	C16I	147.18	6.07	0.04
U16I	U15I	65.48	17.82	0.28
U16I	C16I	7.91	3.51	0.39
C16I	U15I	28.65	19.20	0.18
C16I	U16I	12.50	7.29	0.19
b. Cross-calibration set validations on mashed root samples				
C16M	U16M	6.81	4.38	0.48
**U16M**	**C16M**	**3.10**	**2.59**	**0.72**

For independent validation of models, the mashed calibration set developed at CIAT was trimmed and divided into calibration and validation sets for the three traits–DMC, TCC and ATBC. Previously, the effect of trimming on the Vis/NIRS data was evaluated by comparing calibrations developed from untrimmed and trimmed sets. The result showed that there was no obvious variation or trend between the trimmed and untrimmed data sets (S3 Table). Using the trimmed calibration and validation sets, models were built using the calibration set with larger number of samples and used to predict an independent validation set with fewer training set (Scenario 1) and conversely, using the validation set to predict the values of the larger set (Scenario 2). The average values from the two scenarios were used for independent calibration and validation of models for the three traits. The use of larger number of calibration (Scenario 1) was slightly higher for DMC and ATBC than TCC (Table 8). This probably highlights the role of calibration size on prediction accuracy. The coefficient of determination for prediction (R^2^_p_) ranged from 76% to 91%. On the average, R^2^_p_ for ATBC was highest (91%) followed by TCC (88%) and DMC (80%). The same pattern was observed in RPD distribution. The standard error of prediction corrected for bias SEP(C) was lowest in ATBC (1.65 μg) and highest in TCC (2.36 μg) while DMC was 1.77 percent. The high R^2^_p_ values (>80%) showed that the handheld Vis/NIRS device could be useful in quality and standardized phenotyping in cassava breeding especially for DMC, TCC and ATBC.

**Table 8:** Independent validation of models for DMC, TCC and ATBC.

Trait	Cal	Val	SEP	SEP(C)	R^2^_p_	SD	RPD
DMC	Cal	Val	1.47	1.46	0.836	3.4	2.3
	Val	Cal	2.10	2.08	0.763	4.14	2.0
TCC	Cal	Val	2.64	2.52	0.859	6.62	2.6
	Val	Cal	2.23	2.19	0.901	6.28	2.9
ATBC	Cal	Val	1.70	1.59	0.908	5.21	3.3
	Val	Cal	1.70	1.71	0.902	4.8	2.8

### Correlations of NIRS analyzed, specific gravity and oven-drying dry matter content (DMC) methods

Compared to the current regression equation used by many breeding programs, DMC = 158.3SG– 142 (R^2^ = 0.84) [14–16], the relationship between DMC and SG obtained from the NRCRI dataset was given as DMC = 67.33SG– 37.03 (R^2^ = 0.23). The correlations among the four different DMC methods showed positive relationships among the different methods (Table 9). The highest correlation (0.98) was between oven-drying method and NIRS-derived DMC on mashed root samples. The correlation between oven-drying method and NIRS-derived values from intact root was also very high (0.95) and similar to the relationship between NIRS on intact and mashed root samples (0.94). There was a moderate correlation (0.49) between DMC by oven-drying and specific gravity methods.

**Table 9:** Correlations among the different DMC methods.

	NIRS_I_	NIRS_M_	DM_V_	DM_G_
NIRS_I_	1			
NIRS_M_	0.94	1		
DM_V_	0.95	0.98	1	
DM_G_	0.54	0.49	0.49	1

NIRS_I_ = DMC by portable NIRS on intact root samples; NIRS_M_ = DMC by portable NIRS on mashed root samples; DM_V_ = DMC by oven method; DM_G_ = DMC by specific gravity method.

Although it is very important to standardize the drying conditions for oven-drying method in different breeding programs, it might be necessary for each system to review the relationship between specific gravity and reference DMC by oven-drying and establish protocols for accurate sampling. The low R^2^ value obtained in this study could be attributed to the sampling protocols, weight and number of roots used for specific gravity measurement [14,16]. Field-based specific gravity and for very large population is usually carried out before peeling and cassava peels have been reported to constitute as high as 7.9% of the root size [16] and could even be higher with soil particles and fibrous neck still attached to the root. This could reduce the reported relation between DMC derived by specific gravity and oven-drying which in most cases was carried out after peeling [16]. On the other hand, the use of Vis/NIRS, could help to address the challenges associated with the existing methods while improving the overall quality of phenotyping in cassava.

## Conclusions

From the results of this study, the choice of mathematical pre-processing is a very important step in developing a robust calibration model and the choice of pre-treatment method might be influenced by sampling methods. Calibration models developed with mashed samples were clearly better than intact root samples although the calibration performance for some of the intact root models were still adequate for screening purposes. Also, since the correlation between DMC analysis on intact and mashed root samples was very high, the Vis/NIRS could be employed for initial screening in the field before further extensive laboratory analyses. However, with improved spectra collection protocols and increasing the number of scanning points per root, we hope to further improve calibration performance from intact root samples given that mashing requires additional resources including time and cost of harvesting. The handheld Vis/NIRS has great potential for standardized and unbiased analyses of traits in cassava breeding. It provides a good alternative for the evaluation and improvement of many novel traits which have been difficult or costly to measure before now. In addition to being a non-destructive analytical tool that only requires minimal sample preparation, the portable NIRS is very useful in direct field analyses and will help reduce sample degradation. When compared to the conventional laboratory methods for DMC and carotenoids in cassava breeding, NIRS technique is rapid and cost-effective. It is a good alternative to quality and unbiased evaluation of traits especially in low-cost breeding programs.

## Supporting information

S1 Datasets All the calibration and validation datasets from NRCRI and CIAT in 2015 and 2016 and from either intact or mashed root samples. (ZIP)

S1 Table Calibration for carotenoids from mashed samples using the entire calibration set from CIAT. (DOCX)

S2 Table Calibrations for additional carotenoids from mashed (a) and intact (b) root samples using common samples (n = 66).
(DOCX)

S3 Table Calibrations for DMC, TCC and ATBC using trimmed and untrimmed ASD spectra. (DOCX)

## References

[1] Lopez A, Arazuri S, Garcia I, Mangado J, Jaren C, Accepted J. Review A REVIEW ON THE APPLICATION OF NEAR-INFRARED SPECTROSCOPY FOR THE ANALYSIS OF POTATOES FOR THE ANALYSIS OF POTATOES. 2013; doi: 10.1021/jf401292j 10.1021/jf401292j 23647358

[2] Stuart BH. Infrared Spectroscopy: Fundamentals and Applications [Internet]. Methods. 2004. doi: 10.1002/0470011149

[3] Manley M. Near-infrared spectroscopy and hyperspectral imaging: non-destructive analysis of biological materials. Chem Soc Rev. 2014;43: 8200–8214. doi: 10.1039/c4cs00062e 25156745 10.1039/c4cs00062e

[4] dos Santos CAT, Lopo M, Páscoa RNMJ, Lopes JA. A Review on the Applications of Portable Near-Infrared Spectrometers in the Agro-Food Industry. Appl Spectrosc. 2013;67: 1215–1233. doi: 10.1366/13-07228 24160873 10.1366/13-07228

[5] Büning-Pfaue H. Analysis of water in food by near infrared spectroscopy. Food Chem. 2003;82: 107–115. doi: 10.1016/S0308-8146(02)00583-6

[6] Lu G, Huang H, Zhang D. Prediction of sweetpotato starch physiochemical quality and pasting properties using near-infrared reflectance spectroscopy. Food Chem. 2006;94: 632–639. doi: 10.1016/j.foodchem.2005.02.006

[7] Cabrera-Bosquet L, Crossa J, von Zitzewitz J, Serret MD, Luis Araus J. High-throughput Phenotyping and Genomic Selection: The Frontiers of Crop Breeding Converge. J Integr Plant Biol. 2012;54: 312–320. doi: 10.1111/j.1744-7909.2012.01116.x 22420640 10.1111/j.1744-7909.2012.01116.x

[8] Jannink J-L, Lorenz AJ, Iwata H. Genomic selection in plant breeding: from theory to practice Brief Funct Genomics. Oxford University Press; 2010;9: 166–177. doi: 10.1093/bfgp/elq001 20156985 10.1093/bfgp/elq001

[9] Lu G, Huang H, Zhang D-P. Application of near-infrared spectroscopy to predict sweetpotato starch thermal properties and noodle quality. J Zhejiang Univ Sci B. 2006;7: 475–81. doi: 10.1631/jzus.2006.B0475 16691642 10.1631/jzus.2006.B0475 PMC1474002

[10] Blanco M, Villarroya I. NIR spectroscopy: a rapid-response analytical tool. TrAC Trends Anal Chem. 2002;21: 240–250. http://dx.doi.org/10.1016/S0165-9936(02)00404-1

[11] Sánchez T, Ceballos H, Dufour D, Ortiz D, Morante N, Calle F, et al Prediction of carotenoids, cyanide and dry matter contents in fresh cassava root using NIRS and Hunter color techniques. Food Chem. 2014;151: 444–451. doi: 10.1016/j.foodchem.2013.11.081 24423555 10.1016/j.foodchem.2013.11.081

[12] Ceballos H, Kawuki RS, Gracen VE, Yencho GC, Hershey CH. Conventional breeding, marker-assisted selection, genomic selection and inbreeding in clonally propagated crops: a case study for cassava. Theor Appl Genet. Springer; 2015;128: 1647–67. doi: 10.1007/s00122-015-2555-4 26093610 10.1007/s00122-015-2555-4 PMC4540783

[13] Asrat S, Yesuf M, Carlsson F, Wale E. Farmers’ Preferences for Crop Variety Traits: Lessons for On-Farm Conservation and Technology Adoption. 2009; Available: http://hdl.handle.net/2077/20091

[14] Fukuda WMG, Guevara CL, Kawuki R, Ferguson ME. Selected morphological and agronomic descriptors for the characterization of cassava. Int Inst Trop Agric. 2010; 19.

[15] Kawano K, Fukuda WMG, Cenpukdee U. Genetic and Environmental Effects on Dry Matter Content of Cassava Root1. Crop Sci. 1987;27: 69 doi: 10.2135/cropsci1987.0011183X002700010018x

[16] Pérez JC, Lenis JI, Calle F, Morante N, Sánchez T, Debouck D, et al Genetic variability of root peel thickness and its influence in extractable starch from cassava (Manihot esculenta Crantz) roots. Plant Breed. Blackwell Publishing Ltd; 2011;130: 688–693. doi: 10.1111/j.1439-0523.2011.01873.x

[17] Sánchez T, Chávez A, Ceballos H, Rodriguez-Amaya D, Nestel P, Ishitani M. Reduction or delay of post-harvest physiological deterioration in cassava roots with higher carotenoid content. J Sci Food Agric. John Wiley & Sons, Ltd.; 2006;86: 634–639. doi: 10.1002/jsfa.2371

[18] Belalcazar J, Dufour D, Andersson MS, Pizarro M, Luna J, Londoño L, et al High-throughput phenotyping and improvements in breeding cassava for increased carotenoids in the roots. Crop Sci. 2016;56: 2916–2925. doi: 10.2135/cropsci2015.11.0701

[19] Ortiz D, Sánchez T, Morante N. Sampling strategies for proper quantification of carotenoid content in cassava breeding. Plant Breed Crop …. 2011; Available: http://r4d.dfid.gov.uk/pdf/outputs/misc_crop/ortiz-et-al.pdf

[20] Campbell MR, Mannis SR, Port HA, Zimmerman AM, Glover D V. Prediction of starch amylose content versus total grain amylose content in corn by near-infrared transmittance spectroscopy. Cereal Chem. 1999;76: 552–557. doi: 10.1094/CCHEM.1999.76.4.552

[21] De Alencar Figueiredo LF, Davrieux F, Fliedel G, Rami JF, Chantereau J, Deu M, et al Development of NIRS equations for food grain quality traits through exploitation of a core collection of cultivated sorghum. J Agric Food Chem. 2006;54: 8501–8509. doi: 10.1021/jf061054g 17061827 10.1021/jf061054g

[22] Arganosa GC, Warkentin TD, Racz VJ, Blade S, Phillips C, Hsu H. Prediction of crude protein content in field peas using near infrared reflectance spectroscopy. Can J Plant Sci. 2006;86: 157–159. Available: http://www.nrcresearchpress.com/doi/pdf/10.4141/P04-195

[23] Pizarro C, Esteban-Díez I, Nistal AJ, González-Sáiz JM. Influence of data pre-processing on the quantitative determination of the ash content and lipids in roasted coffee by near infrared spectroscopy. Anal Chim Acta. 2004;509: 217–227. doi: 10.1016/j.aca.2003.11.008

[24] Rinnan Å, Berg F van den, Engelsen SB. Review of the most common pre-processing techniques for near-infrared spectra. TrAC—Trends in Analytical Chemistry. 2009 pp. 1201–1222. doi: 10.1016/j.trac.2009.07.007

[25] Blanco M, Coello J, Iturriaga H, Maspoch S, De La Pezuela C. Effect of data preprocessing methods in near-infrared diffuse reflectance spectroscopy for the determination of the active compound in a pharmaceutical preparation. Appl Spectrosc. 1997;51: 240–246. doi: 10.1366/0003702971939947

[26] Ceballos H, Morante N, Sánchez T, Ortiz D, Aragón I, Chávez AL, et al Rapid cycling recurrent selection for increased carotenoids content in cassava roots. Crop Sci. 2013;53: 2342–2351. doi: 10.2135/cropsci2013.02.0123

[27] Rodriguez-Amaya D., Kimura M. HarvestPlus Handbook for Carotenoid Analysis. Harvest Tech Monogr. IFPRI E-BRARY; 2004; 59 Available: http://ebrary.ifpri.org/utils/getfile/collection/p15738coll2/id/125148/filename/125149.pdf

[28] ASD. ViewSpec Pro TM User Manual. ASD Doc 600555 Rev A. 2008; Available: http://support.asdi.com/Document/Viewer.aspx?id=31

[29] Barnes RJ, Dhanoa MS, Lister SJ. Standard Normal Variate Transformation and De-trending of Near-Infrared Diffuse Reflectance Spectra. Appl Spectrosc. SAGE PublicationsSage UK: London, England; 1989;43: 772–777. doi: 10.1366/0003702894202201

[30] Geladi P, MacDougall D, Martens H. Linearization and scatter-correction for NIR reflectance spectra of meat. Appl Spectrosc. 1985;39: 491–500.

[31] Li D, Liu Y, Chen Y, Wang X, Zhou G. Study on Pretreatment Algorithm of Near Infrared Spectroscopy. 2011; 623–632. Available: http://download.springer.com/static/pdf/977/chp%253A10.1007%252F978-3-642-18336-2_76.pdf?originUrl=http%3A%2F%2Flink.springer.com%2Fchapter%2F10.1007%2F978-3-642-18336-2_76&token2=exp=1495487737~acl=%2Fstatic%2Fpdf%2F977%2Fchp%25253A10.1007%25252F978-3-64

[32] Marten G, Shenk J, Barton F. Near infrared reflectance spectroscopy (NIRS): analysis of forage quality. US Dep Agric Agric Handb. 1989;643: 1–110.

[33] Shenk JS, Westerhaus MO. Population Definition, Sample Selection, and Calibration Procedures for Near Infrared Reflectance Spectroscopy. Crop Sci. 1991;31: 469 doi: 10.2135/cropsci1991.0011183X003100020049x

[34] Freschet GT, Barthès BG, Brunet D, Hien E, Masse D. Use of Near Infrared Reflectance Spectroscopy (NIRS) for Predicting Soil Fertility and Historical Management. Commun Soil Sci Plant Anal. 2011;42: 1692–1705. doi: 10.1080/00103624.2011.584597

[35] Naes T, Isaksson T, Fearn T, Davies T. A User-friendly Guide to Multivariate Calibration and Classification. NIR Publ. 2002;46: 7–289. doi: 10.1198/004017004000000167

[36] Stevens A, Ramirez Lopez L. An introduction to the prospectr package. 2013; 1–22. Available: https://cran.r-project.org/web/packages/prospectr/vignettes/prospectr-intro.pdf

[37] Williams P, Sobering D. Comparison of commercial near infrared transmittance and reflectance instruments for analysis of whole grains and seeds. J Near Infrared Spectrosc. 1993;1: 25–32. doi: 10.1255/jnirs.3

[38] Davrieux F, Dufour D, Dardenne P, Belalcazar J, Pizarro M, Luna J, et al LOCAL regression algorithm improves near infrared spectroscopy predictions when the target constituent evolves in breeding populations. J Near Infrared Spectrosc. 2016;24: 109–117. doi: 10.1255/jnirs.1213

[39] Tillmann P, Reinhardt T-C, Paul C. Networking of near infrared spectroscopy instruments for rapeseed analysis: a comparison of different procedures. J Near Infrared Spectrosc. 2000;8: 103–107. Available: http://journals.sagepub.com/doi/pdf/10.1255/jnirs.269

[40] Terhoeven-Urselmans T. Usefulness of near infrared spectroscopy to assess the composition and properties of soil, litter and growing media Kassel Univ. Press; 2007.

[41] Wang Z, Kawamura K, Sakuno Y, Fan X, Gong Z, Lim J. Retrieval of Chlorophyll-a and Total Suspended Solids Using Iterative Stepwise Elimination Partial Least Squares (ISE-PLS) Regression Based on Field Hyperspectral Measurements in Irrigation Ponds in Higashihiroshima, Japan. Remote Sens. Multidisciplinary Digital Publishing Institute; 2017;9: 264 doi: 10.3390/rs9030264

[42] Fox GP, O’Donnell NH, Stewart PN, Gleadow RM. Estimating hydrogen cyanide in forage sorghum (Sorghum bicolor) by near-infrared spectroscopy. J Agric Food Chem. 2012;60: 6183–6187. doi: 10.1021/jf205030b 22594883 10.1021/jf205030b

[43] Roggo Y, Chalus P, Maurer L, Lema-Martinez C, Edmond A, Jent N. A review of near infrared spectroscopy and chemometrics in pharmaceutical technologies. J Pharm Biomed Anal. 2007;44: 683–700. doi: 10.1016/j.jpba.2007.03.023 17482417 10.1016/j.jpba.2007.03.023

[44] D’Acqui LP, Pucci A, Janik LJ. Soil properties prediction of western Mediterranean islands with similar climatic environments by means of mid-infrared diffuse reflectance spectroscopy. Eur J Soil Sci. Blackwell Publishing Ltd; 2010;61: 865–876. doi: 10.1111/j.1365-2389.2010.01301.x

[45] Pasquini C. Near Infrared Spectroscopy: fundamentals, practical aspects and analytical applications. J Braz Chem Soc. Brazilian Chemical Society; 2003;14: 198–219. doi: 10.1590/S0103-50532003000200006

