# Supplementary material for: Rapid analyses of dry matter content and carotenoids in fresh cassava roots using a portable visible and near infrared spectrometer (Vis/NIRS)
Source: PLoS One. 2017 Dec 11;12(12):e0188918. doi: 10.1371/journal.pone.0188918 (PMC5724885; doi:10.1371/journal.pone.0188918)
Supplement: S1 Table — (DOCX) [file pone.0188918.s002.docx]

S1 Table: Calibration for carotenoids from mashed samples using the entire calibration set from CIAT

| Cal.  set | Traits  (µg) | No. | Range | Mean | SD | SEC | R^2^_c_ | SECV | R^2^_cv_ | RPD |
| --- | --- | --- | --- | --- | --- | --- | --- | --- | --- | --- |
| C16M | VIO | 164 | 0.09-0.92 | 0.47 | 0.17 | 0.042 | 0.94 | 0.11 | 0.61 | 1.55 |
|  | LUT | 100 | 0.02-1.27 | 0.36 | 0.32 | 0.11 | 0.88 | 0.24 | 0.42 | 1.33 |
|  | 15CBC | 163 | 0.01-0.44 | 0.23 | 0.10 | 0.01 | 0.99 | 0.04 | 0.83 | 2.50 |
|  | 13CBC | 165 | 0.04-2.46 | 1.22 | 0.59 | 0.06 | 0.99 | 0.28 | 0.78 | 2.11 |
|  | AC | 74 | 0.03-0.10 | 0.07 | 0.02 | 0.01 | 0.80 | 0.01 | 0.65 | 2.00 |
|  | 9CBC | 171 | 0.10-2.51 | 0.98 | 0.51 | 0.06 | 0.99 | 0.24 | 0.77 | 2.13 |
|  | PHY | 87 | 0.96-13.79 | 5.85 | 2.71 | 0.80 | 0.91 | 2.07 | 0.41 | 1.31 |
