# Supplementary material for: Rapid analyses of dry matter content and carotenoids in fresh cassava roots using a portable visible and near infrared spectrometer (Vis/NIRS)
Source: PLoS One. 2017 Dec 11;12(12):e0188918. doi: 10.1371/journal.pone.0188918 (PMC5724885; doi:10.1371/journal.pone.0188918)
Supplement: S2 Table — Calibrations for additional carotenoids from mashed (a) and intact (b) root samples using common samples (n = 66). (DOCX) [file pone.0188918.s003.docx]

S2 Table: Calibrations for additional carotenoids from mashed (a) and intact (b) root samples using common samples (n=66)

| Cal. set | Traits | No. | Range | Mean | SD | SEC | R^2^_c_ | SECV | R^2^_cv_ | RPD |
| --- | --- | --- | --- | --- | --- | --- | --- | --- | --- | --- |
| 1. Calibration of carotenoids on mashed samples | | | | | | | | | | |
| C16M66 | VIO | 62 | 0.22-0.84 | 0.52 | 0.14 | 0.02 | 0.98 | 0.10 | 0.47 | 1.40 |
|  | LUT | 51 | 0.04-2.36 | 0.65 | 0.54 | 0.20 | 0.87 | 0.44 | 0.33 | 1.23 |
|  | 15CBC | 64 | 0.18-0.44 | 0.29 | 0.06 | 0.02 | 0.91 | 0.04 | 0.60 | 1.50 |
|  | 13CBC | 63 | 0.72-2.42 | 1.57 | 0.40 | 0.08 | 0.96 | 0.22 | 0.68 | 1.82 |
|  | AC | 35 | 0.04-0.10 | 0.07 | 0.02 | 0.008 | 0.73 | 0.01 | 0.47 | 2.00 |
|  | 9CBC | 63 | 0.44-2.04 | 1.22 | 0.36 | 0.07 | 0.96 | 0.21 | 0.65 | 1.71 |
|  | PHY | 63 | 0.96-13.79 | 5.86 | 3.01 | 0.77 | 0.93 | 2.39 | 0.36 | 1.26 |
| 1. Calibration of carotenoids on intact root samples | | | | | | | | | | |
| C16M66 | VIO | 60 | 0.22-0.84 | 0.52 | 0.14 | 0.07 | 0.71 | 0.11 | 0.38 | 1.27 |
|  | LUT | 46 | 0.04-1.32 | 0.51 | 0.34 | 0.30 | 0.22 | 0.32 | 0.10 | 1.06 |
|  | 15CBC | 62 | 0.18-0.44 | 0.29 | 0.06 | 0.02 | 0.88 | 0.04 | 0.44 | 1.50 |
|  | 13CBC | 64 | 0.72-3.05 | 1.59 | 0.43 | 0.22 | 0.73 | 0.37 | 0.27 | 1.16 |
|  | AC | 34 | 0.04-0.10 | 0.08 | 0.01 | 0.01 | 0.67 | 0.01 | 0.46 | 1.00 |
|  | 9CBC | 64 | 0.44-2.04 | 1.22 | 0.36 | 0.15 | 0.82 | 0.29 | 0.34 | 1.24 |
|  | PHY | 61 | 0.96-13.79 | 5.69 | 2.91 | 0.40 | 0.98 | 1.97 | 0.53 | 1.48 |
