# Supplementary material for: Rapid analyses of dry matter content and carotenoids in fresh cassava roots using a portable visible and near infrared spectrometer (Vis/NIRS)
Source: PLoS One. 2017 Dec 11;12(12):e0188918. doi: 10.1371/journal.pone.0188918 (PMC5724885; doi:10.1371/journal.pone.0188918)
Supplement: S3 Table — (DOCX) [file pone.0188918.s004.docx]

S3 Table: Calibrations for DMC, TCC and ATBC using trimmed and untrimmed ASD spectra

| Traits | Sets | Status | SEC | R^2^_c_ | SECV | R^2^_cv_ | SD | RPD |
| --- | --- | --- | --- | --- | --- | --- | --- | --- |
| DMC | Cal. | Untrimmed | 0.49 | 0.987 | 0.93 | 0.953 | 4.34 | 4.7 |
|  |  | Trimmed | 0.63 | 0.979 | 0.91 | 0.956 | 4.36 | 4.8 |
|  | Val. | Untrimmed | 0.37 | 0.990 | 0.93 | 0.937 | 3.75 | 4.0 |
|  |  | Trimmed | 0.33 | 0.993 | 0.87 | 0.946 | 3.79 | 4.4 |
| TCC | Cal. | Untrimmed | 0.58 | 0.994 | 1.94 | 0.928 | 7.24 | 3.7 |
|  |  | Trimmed | 0.51 | 0.995 | 1.78 | 0.937 | 7.14 | 4.0 |
|  | Val. | Untrimmed | 0.90 | 0.985 | 2.28 | 0.897 | 7.20 | 3.2 |
|  |  | Trimmed | 1.15 | 0.972 | 2.41 | 0.875 | 6.89 | 2.9 |
| ATBC | Cal. | Untrimmed | 0.54 | 0.990 | 1.31 | 0.943 | 5.48 | 4.2 |
|  |  | Trimmed | 0.50 | 0.991 | 1.35 | 0.937 | 5.40 | 4.0 |
|  | Val. | Untrimmed | 0.64 | 0.987 | 1.68 | 0.905 | 5.51 | 3.3 |
|  |  | Trimmed | 1.21 | 0.955 | 2.09 | 0.865 | 5.74 | 2.7 |
